# Upregulated TNF/Eiger signaling mediates stem cell recovery and tissue homeostasis during nutrient resupply in *Drosophila* testis

**DOI:** 10.1038/s41598-020-68313-7

**Published:** 2020-07-15

**Authors:** Yi Chieh Chang, Hsin Tu, To-Wei Huang, Bo-Wen Xu, Haiwei Pi

**Affiliations:** 1 Department of Biomedical Sciences, School of Medicine, Chang Gung University, Taoyuan, Taiwan; 2 Department of Psychiatry, Chang Gung Memorial Hospital, Taoyuan, Taiwan

**Keywords:** Developmental biology, Germline development

## Abstract

Stem cell activity and cell differentiation is robustly influenced by the nutrient availability in the gonads. The signal that connects nutrient availability to gonadal stem cell activity remains largely unknown. In this study, we show that tumor necrosis factor Eiger (Egr) is upregulated in testicular smooth muscles as a response to prolonged protein starvation in *Drosophila* testis. While Egr is not essential for starvation-induced changes in germline and somatic stem cell numbers, Egr and its receptor Grindelwald influence the recovery dynamics of somatic cyst stem cells (CySCs) upon protein refeeding. Moreover, Egr is also involved in the refeeding-induced, ectopic expression of the CySC self-renewal protein and the accumulation of early germ cells. Egr primarily acts through the Jun N-terminal kinase (JNK) signaling in *Drosophila*. We show that inhibition of JNK signaling in cyst cells suppresses the refeeding-induced abnormality in both somatic and germ cells. In conclusion, our study reveals both beneficial and detrimental effects of Egr upregulation in the recovery of stem cells and spermatogenesis from prolonged protein starvation.

## Introduction

Tissue homeostasis relies on precise regulation of stem cell activity and cellular differentiation. Identifying the signals that connect environmental stimuli to specific stem cells and their microenvironment is crucial in deciphering the mechanism for tissue homeostasis maintenance.

Germline is actively maintained by germline stem cells (GSCs). Germline and GSC homeostasis is robustly influenced by nutrient availability. In *C .elegans*, prolonged nutritional deprivation induces reproductive diapause, eliminating entire germline except for a small population of GSCs through apoptosis. Resumed nutrient uptake regenerates new germline from the protected GSCs^1^. In female *Drosophila,* egg production is dramatically reduced under the protein-poor diet^2^. This is partly due to the reduced division rate of both GSCs and follicle stem cells, and their progeny. In addition, cell death of germ cell cysts is also increased by low protein diet.

In male *Drosophila* under chronic protein starvation, GSC numbers are maintained by nutrients released from the dying spermatogonia. Starvation-induced spermatogonia death is triggered by the apoptosis of the surrounding somatic cyst cells^3^, which enclose the germ cells to regulate their self-renewal, proliferation and differentiation^4–8^. Protein resupply recovers GSC numbers and division, at least in part due to JNK-mediated de-differentiaion of spermatogonia to GSCs^9^. Somatic cyst cells are generated from division of cyst stem cells (CySCs). The population of early cyst cells, including CySCs, is reduced by half during prolonged protein starvation, and recovers upon protein refeeding^10^. However, the mechanism for the cyst lineage cell recovery through nutrient resupply remains largely unknown.

The *Drosophila* testis is surrounded by a sheath composed of an outer layer of pigment cells and the inner layer of smooth muscles. The smooth muscles lie immediately adjacent to cyst cells with a thin layer of extracellular matrix between them^11^. Recent studies have shown that testicular muscles produce tumor necrosis factor (TNF) to influence spermatogenesis^12^. Eiger (Egr) is the sole *Drosophila* TNF homologue. It was first discovered by its potent activity to induce apoptosis^13–15^. Although it is not essential for normal development, Egr plays critical role in the maintenance of *Drosophila* physiology and vitality in response to pathogen infection^16^, harmful environmental stimuli, and injury^17–19^. Egr induces cellular responses by binding to the TNF receptors (TNFRs), Wengen and Grindelwald (Grnd)^20,21^. Unlike mammalian TNFs that activate NF-κB, MAPK and JNK pathways, Egr-induced cellular responses are mainly mediated by JNK signaling activation only^22^.

Previously we have found that TNF-JNK signaling is hyper-activated by reproduction in testes, leading to ectopic expression of the CySC self-renewal protein Zfh-1^12^. In this study, we show that prolonged protein starvation induces Egr upregulation in testis smooth muscle. Upon protein resupply, Egr and the receptor Grnd are required for the rapid recovery of CySC population. In addition, a novel phenotype in germline recovering from prolonged protein shortage is discovered; Zfh-1 is ectopically expressed in differentiating cyst cells away from the apical niche, resulting in overproduction of early germ cells. Both *egr* depletion in testis smooth muscles and JNK signaling inactivation in cyst cells suppress the abnormalities, indicating that too much TNF induced by prolonged protein starvation disrupts the germline recovery via JNK signaling in cyst cells. Moreover, we found that prolonged protein starvation impairs proteasome activity in testicular muscle. Inactivation of proteasome functions fails to increase Egr levels, suggesting that additional mechanism is required to upregulate TNF in smooth muscle during starvation.

## Results

### Egr is induced in testicular smooth muscles during protein starvation

**Figure 1:**
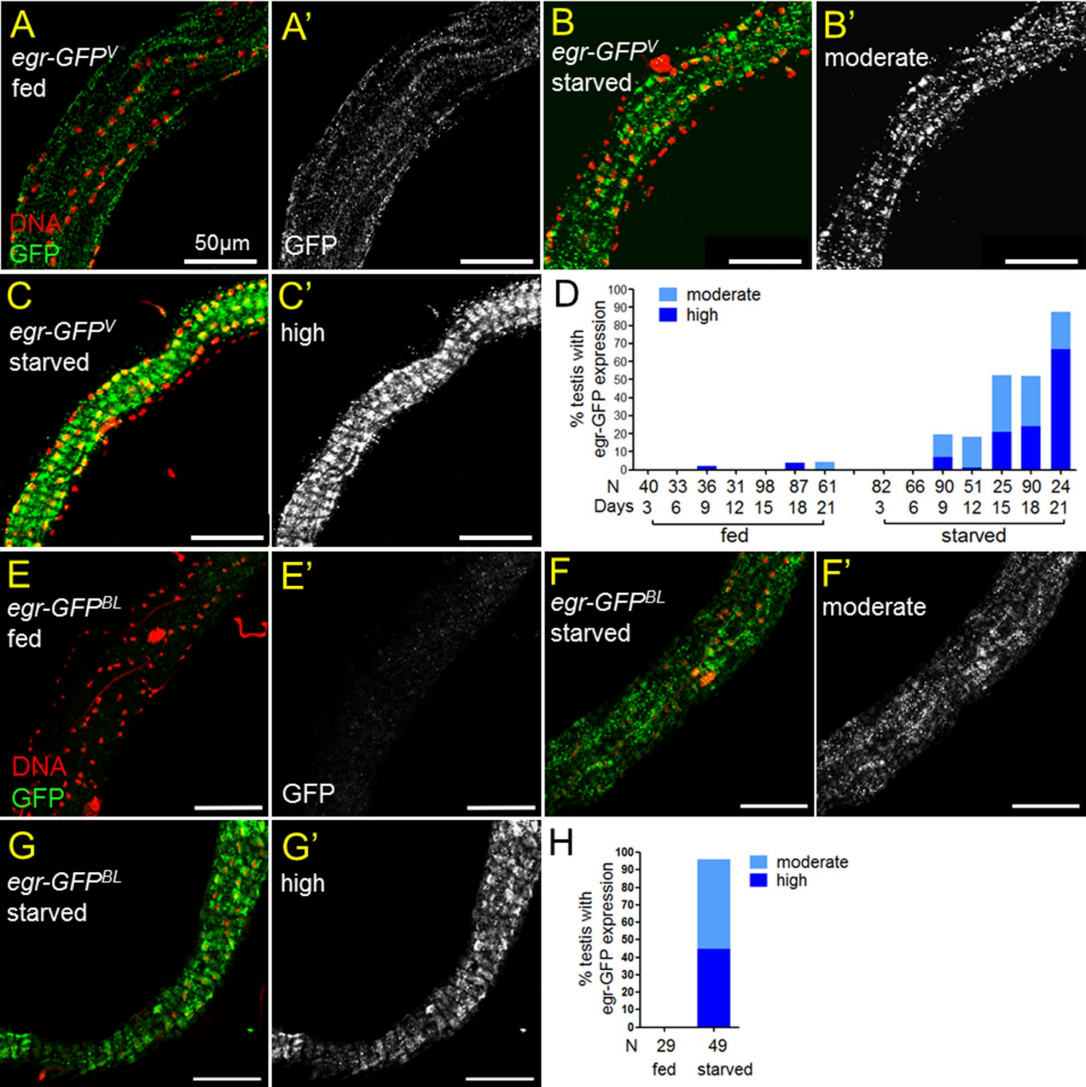
Protein starvation upregulates Egr levels in testicular muscles. (**A**–**C**’ and **E**–**G**’) Testes of *egr-GFP*^*v*^ (**A**–**C**’) and *egr-GFP*^*BL*^ (**E**–**G**’) immunostained for GFP (green in **A**–**C** and **E**–**G**, and white in **A**’–**C**’ and **E**’–**G**’). Nuclei are labeled by Hoechst 33342 (red). Flies were either raised on standard protein-rich food (fed) or protein-restrict diet (starved) for 18 days. Egr-GFP signals were detected in testicular muscles from starved males (**B**,**C**,**F**,**G**), but not from fed males (**A**,**E**). The grade of Egr-GFP expression was classified to moderate (**B**,**B**’ and **F**,**F**’) or high (**C**,**C**’ and **G**,**G**’) based on the signal intensity and the size of GFP-positive area (see “Methods”). Scale bars 50 μm. (**D**,**H**) Percentages of testes with detectable Egr-GFP expression in the testicular muscles. (**D**) The percentages of testes expressing Egr-GFP in smooth muscles were increasing over time in males starved from 9 to 21 days. (**H**) GFP accumulation in smooth muscles was detected in almost all testes from *egr-GFP*^*BL*^ males starved for 18 days. GFP was not detected in the testicular muscles of males fed for 18 days.

Previously we have shown that reproduction increases Egr levels in the testicular muscle to hyperactivate JNK signaling in somatic cyst cells^12^. Since JNK signaling is also activated in cyst cells in response to amino acid starvation^3^, we then asked if protein starvation induces Egr protein upregulation in testicular smooth muscles. By examining the levels of Egr-GFP expressed from a genomic fosmid clone (referred as *Egr-GFP*^*v*^)^23^, upregulation of GFP in smooth muscles was specifically observed in testes from flies suffered from prolonged protein starvation (starved) (Fig. 1B–D). Egr-GFP was not observed in the testicular muscles of males starved for 3 and 6 days. However, more than 50% and 80% of the testes from flies starved for 15 days and 21 days, respectively, had increased Egr levels in the testicular muscles (Fig. 1D). Egr-GFP was almost not detected in the testis muscle of 3- to 21-day old males fed with protein-containing food (fed) (Fig. 1A, D). To confirm the upregulation of Egr in response to protein starvation, we examined an *Egr-GFP* protein trap line in which GFP is inserted near the transmembrane domain of the endogenous Egr (referred as *egr-GFP*^*BL*^)^24^. As shown in Fig. 1E–H, GFP was highly expressed in the testicular smooth muscles of *egr-GFP*^*BL*^ males starved for 18 days, but not in the testis muscles of the age-matched fed males. Together, we conclude that Egr protein level is upregulated in testicular smooth muscle in response to prolonged amino acid starvation.

### Protein starvation impairs proteasome activity in testicular muscles

**Figure 2:**
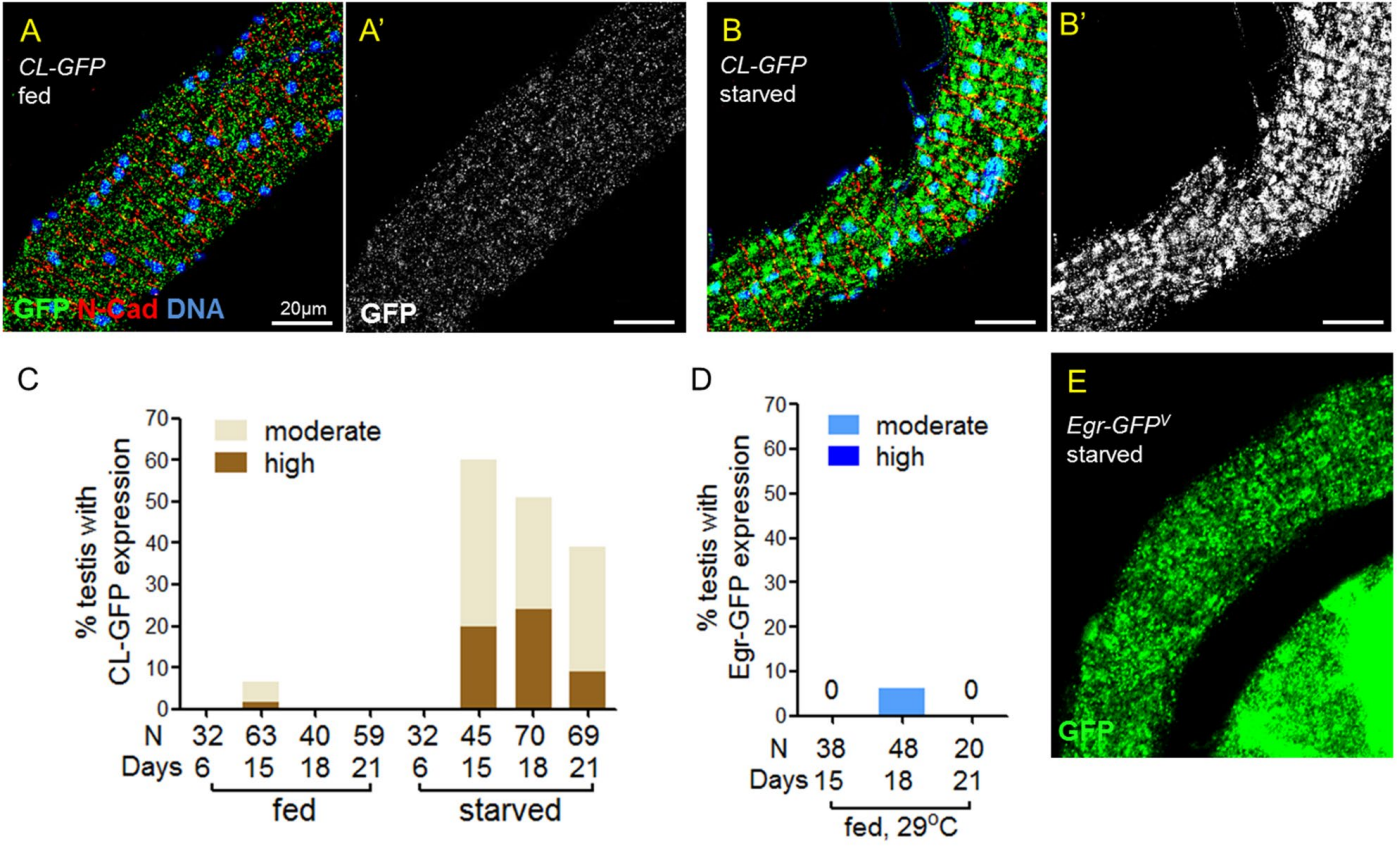
Protein starvation impairs ubiquitin–proteasome activity in testis muscle. (**A**–**B**’) Testes from *mef2* > *CL1-GFP* flies immunostained for GFP (green), co-stained for N-cadherin (red) and DNA (blue). Anti-N-cadherin staining labels the muscle membrane. Flies were either fed (**A**,**A**’) or starved (**B**,**B**’) for proteins for 18 days. (**C**) The percentages of testes showing CL1-GFP accumulation in the testicular muscles. Testes were from males either fed or starved for 6, 15, 18 and 21 days. (**D**,**E**) GFP expressed from *Egr-GFP*^*v*^ in testes of *mef2* > *DTS5 DTS7* males. (**D**) The percentage of testes showing GFP accumulation in the muscles. Fed flies were maintained at 29 °C to enhance DTS5 and DTS7 expression for 15, 18 or 21 days as indicated. (**E**) Testes immunostained for GFP. Upregulation of Egr-GFP was not detected in testicular muscle until starved for 9 days. Adult flies were maintained at 29C during the starvation phase.

Proteasome-mediated degradation is the major mechanism for protein turnover. The Egr-GFP accumulation prompts us to ask whether protein starvation impairs proteasome functions in testicular muscles. To answer this question, we examined the abundance of the ubiquitin–proteasome system reporter CL1-GFP that carries the degron CL1^25^. In contrast to almost undetectable GFP signal in animals fed with normal food for 6–21 days (Fig. 2A, C), CL1-GFP was detected abundantly in testicular muscle in males starved for 15–21 days (Fig. 2B, C), indicating that prolonged protein starvation impairs ubiquitin–proteasome system in the testis muscles.

To further test whether decreased proteasome activity contributes to Egr-GFP accumulation, proteasome activity was disrupted by overexpression of the dominant, temperature-sensitive proteasome mutants *DTS5* and *DTS7*^26^. The presence of DTS5 at non-permissive temperature (29 °C) for 24 h is sufficient to disrupt proteasome activity^26^. To our surprise, overexpression of *DTS5* and *DTS7* in muscles at 29 °C for 15–21 days did not lead to detectable Egr-GFP expression in testicular smooth muscles in fed males (Fig. 2D). In starved males expressing *DTS5* and *DTS7* at 29 °C in the muscles, Egr-GFP was not observed in testicular muscle until starvation for nine days (Fig. 2E), at that time when starved males without *DTS5* and *DTS7* expression also began to produce Egr-GFP in the muscle (Fig. 1D). Therefore, our results suggest that compromised ubiquitin–proteasome activity in testicular muscle by protein starvation is not the major mechanism for Egr upregulation.

### Egr does not affect stem cell loss during protein starvation

**Figure 3:**
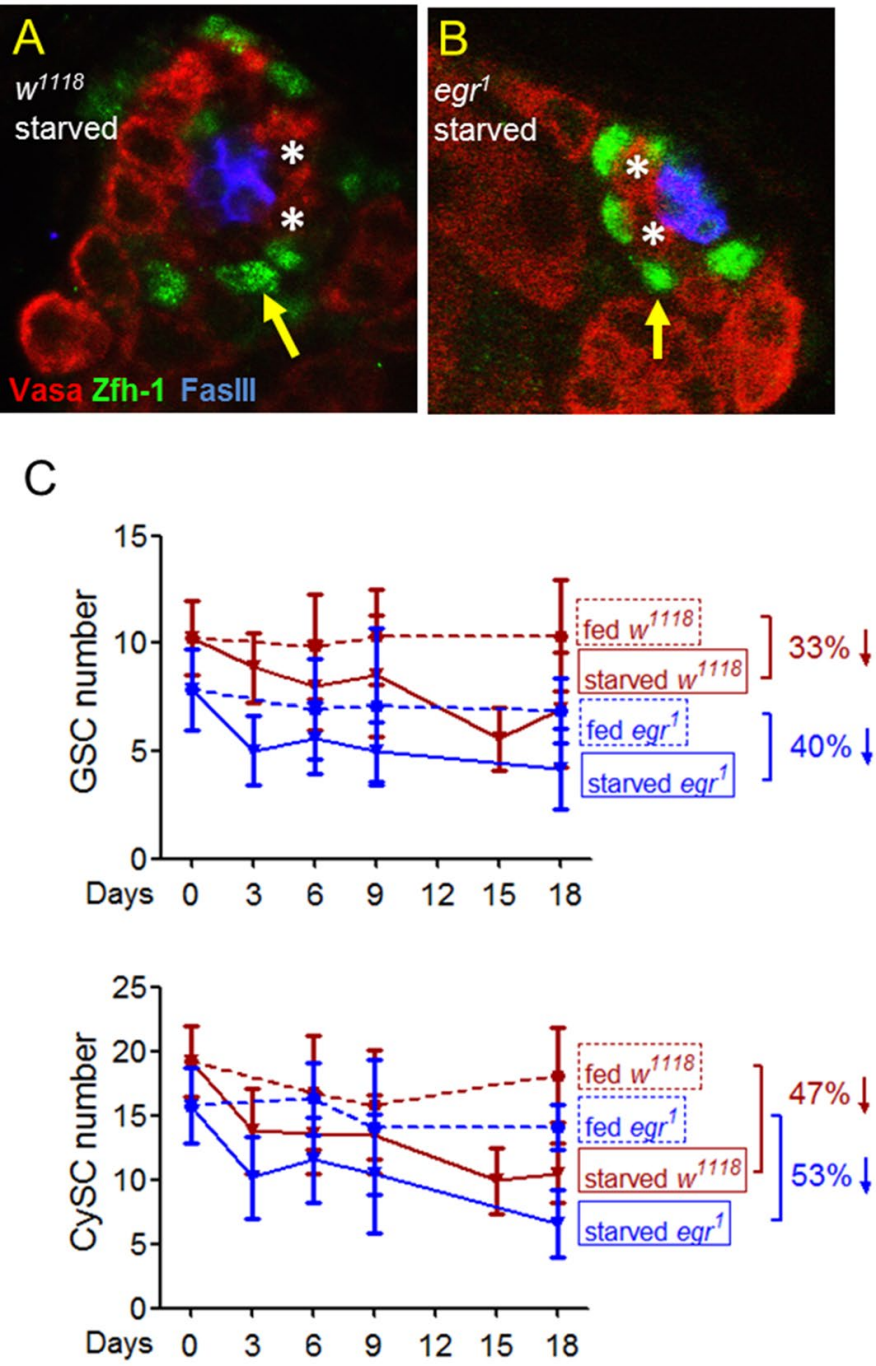
*egr* is not essential to regulate stem cell numbers in response to protein starvation. (**A**,**B**) Testes immunostained for Vasa (germ cells, red), co-stained for Zfh-1 (CySCs and early cyst cells, green) and FasIII (hub, blue). Asterisks and arrows indicate GSCs and CySCs, respectively. Flies were starved from 18 days. (**C**) The number of GSC and CySC after starvation for 0–18 days. Data is presented as mean ± SD. The number of testes in each time point is between 15 and 34.

Since Egr-induced cellular responses are primarily mediated by JNK signaling, and JNK activation in cyst cells is required to maintain GSC numbers during protein starvation^3^, we first examined GSC numbers in *egr* mutants starved for amino acids. In testes of *w*^*1118*^ controls starved for 18 days, GSC number was reduced 33% (10.4 ± 2.6 vs. 6.9 ± 2.7) compared to the age-matched fed adults (Fig. 3A and upper panel in 3C)^3,10^. Starvation caused 40% decrease in GSC number in *egr*^*1*^ null mutants (6.9 ± 1.5 vs. 4.2 ± 1.9) (Fig. 3B and upper panel in 3C). The reduction of CySC number by amino acid starvation was also comparable between *egr*^*1*^ (53%, 14.1 ± 1.7 to 6.6 ± 2.7) and the control (47%, 18.1 ± 3.7 to 10.5 ± 2.3) (Fig. 3A, B, and bottom panel in 3C). Therefore, *egr* appears not critical for reduction or maintenance of somatic and germline stem cells by protein starvation.

### Lack of *egr* delays CySC recovery from starvation

**Figure 4:**
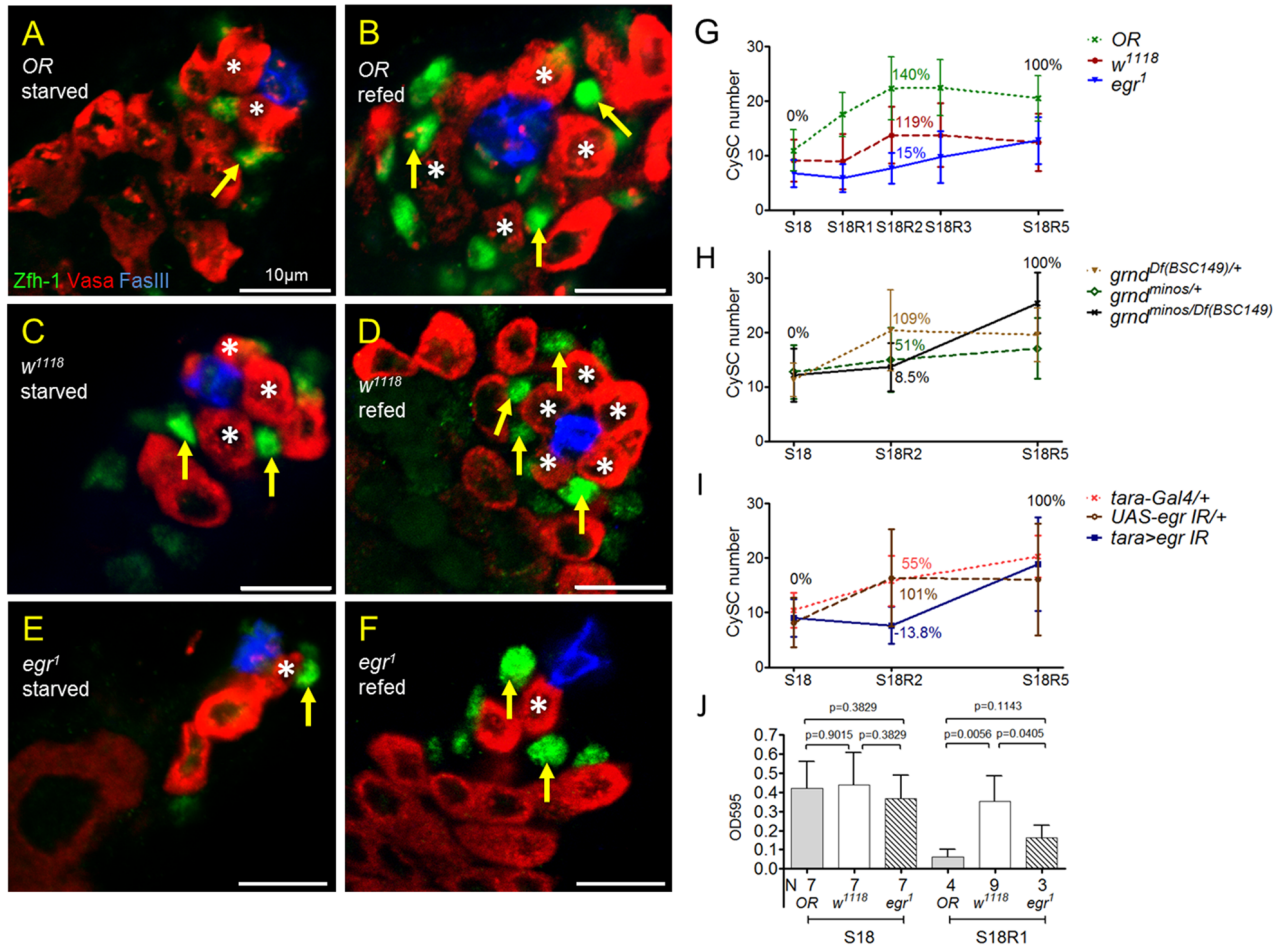
Egr is required for fast recovery of CySC number upon protein refeeding. (**A**–**F**) Testes immunostained for Zfh-1 (green), co-stained for Vasa (red) and FasIII (blue). (**A**,**C**,**E**) Testes from males starved for 18 days (S18). (**B**,**D**,**F**) Testes from males starved for 18 days and refed for two days (S18R2). (**G**–**I**) Average number of CySCs during refeeding phase. Data are presented as mean ± SD. The CySC recovery rate at S18 and S18R5 is set as 0% and 100%, respectively. The recovery rate at R2 is indicated (see “Methods”). (**G**) CySC recovery in *Oregon R* (*OR*), *w*^*1118*^ and *egr*^*1*^ adults upon refeeding. Number of testes for each time point is between 11 and 36. (**H**) Lack of Grnd in *grnd*^*minos*^*/Df (BSC149)* mutant delayed CySC recovery upon refeeding. The number of testes for each point is between 25 and 38. (**I**) Tissue-specific depletion of *egr* in testicular muscle delayed CySC recovery upon refeeding. The number of testes for each point is between 19 and 37. (**J**) Quantification of food consumed by adult flies after starvation for 18 days (S18) and 1-day refeeding (S18R1). Statistics were generated by Student *t* test.

The reduction in the number of stem cells by starvation is reversible upon refeeding^10^. We found that Egr is required for the fast recovery of CySCs induced by protein resupply. CySCs are defined as the Zfh-1 positive cells immediately adjacent to GSCs^12,27^. In wild-type *Oregon-R* (*OR*) flies, the number of CySC increased dramatically from 11 to 22 during the first two days of refeeding (R1 and R2) (compare Fig. 4B to 4A, and 4G). Because there was slight decrease in CySC number from refeeding day 3 to day 5 (R3–R5) (Fig. 4G), the CySC number at R2 was the highest during the five-day refeeding phase. The recovery rate was 140% at R2 compared to R5. In *w*^*1118*^, the number of CySCs also reached to the highest after two days of refeeding. The recovery rate at R2 was 119% compared to that at R5 (compare Fig. 4D to 4C and 4G). In *egr*^*1*^, however, CySC recovered much slower. The CySC recovery rate at R2 was 15% to that at R5 (compare Fig. 4F to 4E, and 4G). CySC number continued to increase gradually each day from R2 to R5 in *egr*^*1*^ adults. Egr acts on cyst lineage cells through the receptor Grnd^12^. Examination of CySC recovery in *grnd* null mutant (*grnd*^*minos*^*/Df*) showed that the recovery rate was very low at R2 compared to R5 (9%, Fig. 4H). Together, our results reveal a late recovery of CySC population in the absence of Egr and Grnd.

Since Egr was expressed in the testicular smooth muscles, we tested whether depletion of *egr* from the muscles influences the dynamics of CySC recovery upon refeeding. Tissue-specific knockdown of *egr* by RNAi driven via *tara-Gal4*, that is specifically expressed in the muscles but not the other cells in the testis^12^, markedly reduced the CySC recovery rate at R2 compared to R5 (− 14%, Fig. 4I). The CySC recovery rate at R2 was much higher in both Gal4 and UAS control flies (55% in Gal4 control, 101% in UAS control,) (Fig. 4I). Thus, our results suggest that Egr produced from testicular muscle is important for the rapid CySC recovery upon refeeding.

It has been shown that *egr* mutant adults ate less food compared to *w*^*1118*^ or several Gal4 lines when fed constantly with protein-containing food^28^. The slower recovery of CySC in *egr*^*1*^ flies during the refeeding period, thus, might be resulted from less food consumption. To answer the question whether CySC recovery rate upon refeeding positively correlates with the ingestion rate, we quantified the food intake. *OR*, *w*^*1118*^ and *egr*^*1*^ flies ingested comparable amount of protein-containing foods after 18 days of amino acid starvation (S18) (Fig. 4J). Interestingly, *OR* flies ingested the least amount of food after refeeding for one day (S18R1) despite the highest recovery rate during the first two days of refeeding (Fig. 4G). The amount of food consumed by *OR* flies was less than 20% of the food by *w*^*1118*^ adults. The *egr*^*1*^ flies consumed more food than *OR* but ingested less than *w*^*1118*^ at R1 (Fig. 4J). Our results suggest that the rate of ingestion does not positively correlate with the rate of CySC recovery from amino acid starvation. Thus, the slow recovery of CySC upon refeeding is unlikely resulted from eating too little in *egr*^*1*^ mutants.

### Protein refeeding leads to dysregulation of Zfh-1 in cyst cells via Egr

**Figure 5:**
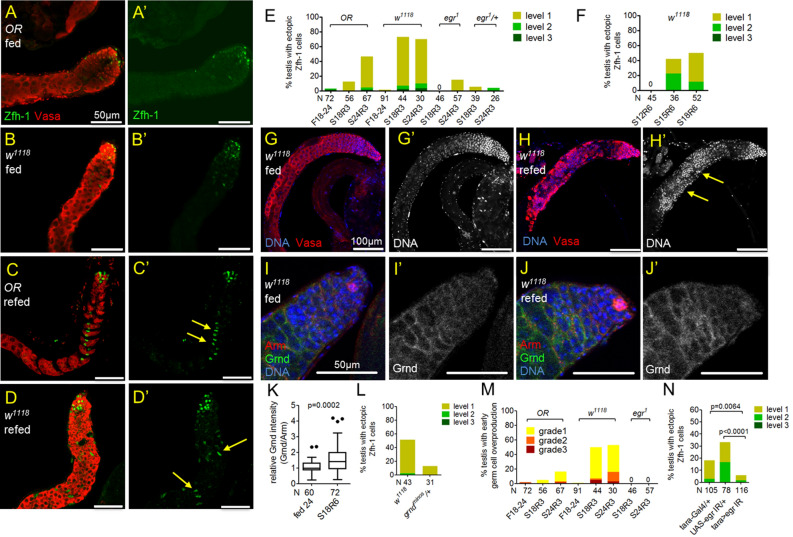
Egr and Grnd are essential for ectopic Zfh-1 expression by protein refeeding. (**A**–**D**’) Testes immunostained for Zfh-1 (green in **A**–**D**’), co-stained for Vasa (red). (**A**–**B**’) Zfh-1-positive cells were restricted to the apical region in testes of *OR* and *w*^*1118*^ flies fed for 24 days. (**C**–**D**’) Ectopic Zfh-1 expression (arrows) outside the apical region was found in testes from *OR* and *w*^*1118*^ males starved for 18 days and re-fed for 6 days. (**E**) Percentages of testes exhibiting ectopic Zfh-1 expression. Refeeding induced ectopic Zfh-1 expression in testes from *OR* and *w*^*1118*^. Ectopic Zfh-1 expression was rarely observed in testes from refed males of *egr*^*1*^ homozygous or heterozygous. Ectopic Zfh-1 expression was almost not observed in males fed for 18–24 days. (**F**) Percentages of testes showing ectopic Zfh-1 expression. Testes were form males starved for 12–18 days, followed by refeeding for 6 days. (**G**–**H**’) Testes immunostained for DNA (blue in **G** and **H**, white in **G**’ and **H**’), co-stained for Vasa (red). Early germ cells characterized by intense DNA staining were overproduced (arrows) in the testes from males starved for 18 days followed by refeeding for 6 days (**H**). Overproduction of early germ cells was not found in testes from males fed for 24 days (**G**). (**I**–**J**’) Testes immunostained for Grnd (green in **I** and **J**, white in **I**’ and **J**’), con-stained for Arm (cyst cell cytoplasm, red) and DNA (blue). Anti-Grnd staining was observed specifically on the cyst cells. (**K**) Box-and-whisker plots showing relative anti-Grnd/anti-Arm signal intensity in cyst cells, normalized to the median value of anti-Grnd/anti-Arm intensity in the testes from fed males. Significantly higher levels of anti-Grnd staining were observed in the re-fed males (S18R6) compared to the age-matched fed males (fed 24). P value was calculated by Mann–Whitney nonparametric test. (**L**) Percentages of testes exhibiting ectopic Zfh-1 expression. One copy of *grnd* null allele (*grnd*^*minos*^) dominantly suppressed the ectopic Zfh-1 expression in the testes of the refed males (S18R6). (**M**) Percentages of the testes with overproduction of early germ cells. Early germ cell overproduction by refeeding was specifically observed in wild-type (*OR* and *w*^*1118*^) males, but not in *egr*^*1*^ mutants. (**N**) Knockdown of *egr* in testis muscles via *tara-Gal4* suppressed ectopic Zfh-1 expression induced by refeeding. Testes are dissected from refed (S18R6) males. *P* value was calculated by Chi-squared test.

Zfh-1 maintains CySC self-renewal in somatic cyst cell lineage^29^. Its expression is normally activated in CySCs and early cyst cells in the apical region via signals from hub located in the testis tip^27,29–32^ (Fig. 5A, A’ and Fig. 5B, B’). Interestingly, Zfh-1 was ectopically expressed in cells away from the hub in wild-type *OR* and *w*^*1118*^ flies refed after prolonged protein starvation (Fig. 5C, C’, D, D’). In *OR* flies starved for 18 or 24 days and refed for 3 days, 13% and 46% testes, respectively, exhibited ectopic Zfh-1 expression away from the apical region (Fig. 5E). Ectopic Zfh-1 expression was also observed in more than 50% of the testes from *w*^*1118*^ males starved for 18 or 24 days and refed for 3 days (Fig. 5E). In contrast, ectopic Zfh-1 positive cells were rarely observed in *OR* and *w*^*1118*^ males fed with protein for 18–24 days (Fig. 5E). The dysregulation of Zfh-1 expression in testes from refed animals was only observed with prolonged starvation. For example, it was not observed in *w*^*1118*^ males starved for 12 days, but was found in 40% and 50% of testes from males starved for 15 days and 18 days, respectively (Fig. 5F).

Cyst cells are critical to instruct germ cell differentiation^5,7,33^. Ectopic Zfh-1 expression in differentiating cyst cells non-autonomously blocks germ cell differentiation, leading to accumulation of early germ cells^12^. Consistently, we observed overproduction of early germ cells, which can be recognized by their smaller cell size and intense DNA staining, in testes from refed animals but not from fed animals (compare Fig. 5H’ to Fig. 5G’, and Fig. 5M).

Since the abnormal phenotypes in testes from refed males were reminiscent to the Egr-dependent Zfh-1 dysregulation by reproduction^12^, we then examined whether *egr* is also involved in the Zfh-1 abnormality induced by refeeding. Loss of *egr* almost completely suppressed the ectopic Zfh-1 expression and the early germ cell overproduction in *egr*^*1*^ mutants (Fig. 5E, M). We also found that refeeding significantly elevated the levels of the receptor Grnd on the cyst cells (Fig. 5I–K). Consistently, the percentages of testes exhibiting ectopic Zfh-1 expression was dominantly suppressed by lack of one copy of *egr* or *grnd*, as compared to the parental control *w*^*1118*^ (Fig. 5E, L). Tissue-specific depletion of *egr* by RNAi via *tara-Gal4* significantly suppressed ectopic Zfh-1 expression in testes from re-fed animals (Fig. 5N). Together, our results strongly suggest that Egr and Grnd are essential for refeeding-induced Zfh-1 dysregulation.

### JNK signaling activation in cyst cells promotes Zfh-1 dysregulation upon refeeding

**Figure 6:**
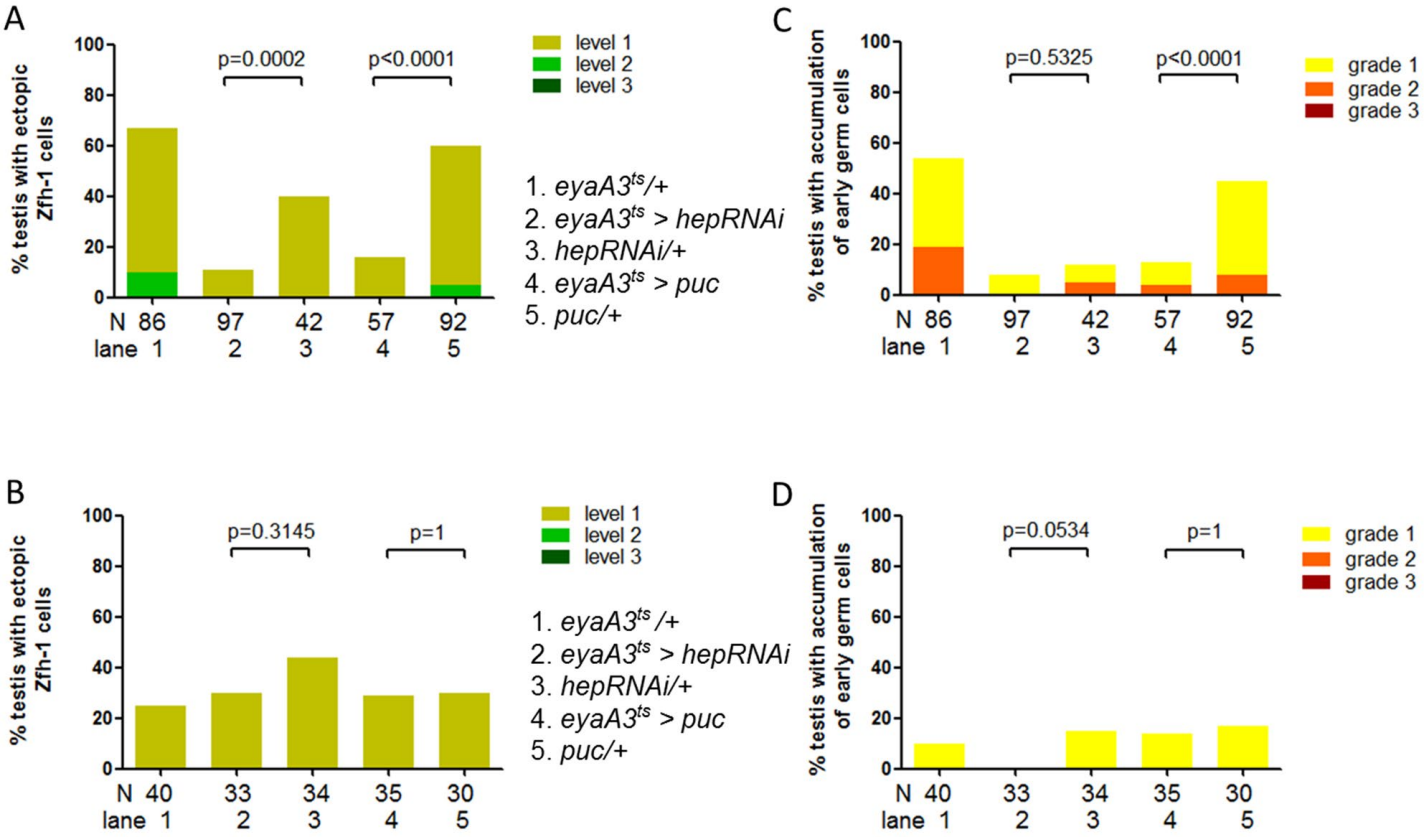
JNK signaling activation is necessary for Zfh-1 dysregulation induced by refeeding. Percentages of testes exhibiting ectopic Zfh-1 expression (**A**, **B**) and early germ cell overproduction (**C**, **D**). (**A**) Ectopic Zfh-1 expression by refeeding was markedly suppressed by depletion of *hep* or overexpression of *puc* in cyst lineage cells at restrictive temperature 29 °C. (**B**) No suppression was observed when Gal80^ts^-mediated JNK signaling inactivation was blocked at permissive temperature 25 °C. (**C**) Refeeding-induced early germ cell overproduction was also noticeably suppressed by *puc* overexpression in cyst lineage cells at 29 °C. (**D**) No significant suppression of early germ cell accumulation was observed at permissive temperature 25 °C. Statistics were generated using Chi-squared test.

Protein refeeding activates JNK signaling in germline and cyst cell lineage^9^. While JNK signaling activation in germline is required for de-differentiation of spermatogonia to GSCs, the role of JNK signaling hyperactivation in cyst lineage is unknown. Because *egr* is critical for refeeding-induced Zfh-1 dysregulation, we tested whether JNK signaling is also involved in it via temperature-sensitive Gal80^ts^-mediated inhibition. It has been shown that depletion of JNKK encoding by *hemipterous* (*hep*) or forced expression of JNK phosphatase encoding by *puckered* (*puc*) suppresses JNK signaling responses^34^. JNK signaling was temporally inactivated in the refeeding phase by temperature shift from 25 to 29 °C three days before and during the entire refeeding phase. Knockdown of *hep* or overexpression of *puc* in cyst lineage cells by *eyaA3-Gal4; tub-Gal80*^*ts*^ (*eyaA3*^*ts*^) markedly suppressed the percentages of testes with ectopic Zfh-1 expression when compared to the controls (Fig. 6A). In contrast, there was no significant suppression in the percentages of testes with extra Zfh-1 cells when JNK signaling inactivation was blocked at 25 °C during the entire starvation and refeeding phases (Fig. 6B). Overexpression of *puc* in cyst lineage also noticeably decreased the percentage of the testes exhibiting early germ cell overproduction at 29 °C but not at 25 °C (compare Fig. 6C and D). Together, our results indicate that protein refeeding induces aberrant Zfh-1 expression and excess early germ cells via JNK signaling activation in cyst lineage cells.

## Discussion

Nutrient availability robustly influences animal physiology. In this study, we show that long-term protein starvation induces Egr production in the testis smooth muscles. While Egr is not essential for loss or maintenance of stem cells by protein starvation, it is required for the fast recovery of somatic cyst stem cells upon refeeding. Furthermore, we also observed that refeeding after chronic protein starvation leads to ectopic Zfh-1 expression and overproduction of early germ cells. The aberrant phenotypes upon refeeding are resulted from TNF-JNK signaling hyper-activation in the somatic cells. Since TNF-JNK signaling hyperactivation induces Zfh-1 expression in normal, nutrient-supply condition^12^, we propose that Egr induced by starvation “primes” cyst cells for Zfh-1 expression upon nutrient resupply, promoting CySC recovery and ectopic Zfh-1 expression in cyst lineage cells during protein refeeding.

We found that prolonged protein starvation promotes Egr upregulation in the testis muscle. Together with our previous observation that reproduction increases Egr levels in testis muscle in aged males, these results suggest that smooth muscle is the sensor tissue of the male gonads to sense the physiological changes. Indeed, many other studies have shown the critical roles of muscle to signal to other tissues for the adaptive responses. In *Drosophila*, Wingless (Wg) proteins from visceral muscle of the gut act non-autonomously on enterocytes to influence gut homeostasis^35^. In both mammals and flies, genetic alteration of signaling events or enzymatic activity in skeletal muscles could modulate stress resistance and lifespan of the organisms, via endocrine or paracrine mechanisms^36^.

It has been postulated that reproduction-induced Egr upregulation in smooth muscle might be resulted from muscle damage by chronic stress and aging^37^. While we observed severely impaired proteolysis in testicular smooth muscle in males suffered from chronic protein starvation, disruption of ubiquitin–proteasome pathway neither induced Egr upregulation in fed males nor enhanced Egr levels in starved males. These results suggest that proteasome-mediated protein turnover plays little role in regulation of Egr in testicular muscle. It is likely that other damages commonly observed in muscles such as accumulated DNA damage^38,39^ or compromised autophagy^40^ might also be required to modulate Egr levels. In *Drosophila*, stress- and nutrient-sensing pathways such as p38 kinase and mTOR signaling are required in skeletal muscles to modulate normal life span and stress responses^41,42^. Alternatively, Egr levels might be regulated by the nutrient- and stress-sensing pathways. Muscle is more sensitive to aging-induced apoptosis and DNA damage than the other tissues in *Drosophila*^38,43^. Interestingly, Egr upregulation in response to starvation and reproduction was only observed in older males. The changes in response to starvation and reproduction must couple with age-associated events to modulate Egr levels in testicular muscles.

Previous works have shown that JNK signaling is activated in cyst cells by protein starvation to promote cyst cell apoptosis, and is activated in germ cells upon protein refeeding to induce germ cell de-differentiation^3,9^. Together with our results, it shows that nutrient availability act through JNK signaling to modulate different tissue responses in adult testes in both starvation and refeeding phases, and in both somatic cells and germline. Our results, however, is the first to discover the mechanisms acting upstream of JNK signaling activation. Although protein starvation induced Egr upregulation and JNK-dependent cyst cell apoptosis, testis involution and comparable stem cell number were still observed in starved *egr* mutants. It suggests that Egr influences testis homeostasis in a context-dependent manner.

It has been shown that amino acid starvation promotes Egr secretion from fat body to activate JNK signaling in brain, that in turn reduces insulin peptide production and inhibits larval growth^44^. However, our studies of adult testis showed that TNF-JNK signaling act locally to modulate cyst cell and germ cell homeostasis upon refeeding. TNFR Grnd level was significantly upregulated in cyst cells in the testes from re-fed males, and reduction of JNK signaling in cyst cells suppressed Zfh-1 dysregulation and overproduction of early germ cells. Protein starvation rapidly induces TACE expression to release soluble Egr from fat body during larval growth^44^. Instead, our results showed that both Egr upregulation in testicular muscles and ectopic Zfh-1 expression in cyst cells were only observed after prolonged starvation. Together, these results suggest diverse and distinct functions of TNF-JNK signaling in modulating systemic body growth of larvae and homeostasis of one particular adult tissue in response to protein starvation.

## Methods

### Stocks and fly husbandry

*Oregon-R* (*OR*) and *w*^*1118*^ were used as wild-type, control strains. *egr*^*1*^ and *grnd*^*minos*^ (BL43677) are null alleles^13, 21^. *Df* (*BSC149*) is the deletion line for *grnd*^21^. *egr-GFP*^*v*^ (v318615) and *egr-GFP*^*BL*^ (BL66381)^24^ are for detection of Egr proteins. UAS-CL1-GFP was used as a reporter for proteome activity as described^45^. *UAS-DTS5* and *UAS-DTS7* are used to disrupt proteasome activity^26^. *eyaA3-Gal4;tub-Gal80*^*ts*^ (referred as *eyaA3*^*ts*^)^29^, *tara-Gal4* (BL-63905)^46^ and *mef2-Gal4* (BL25765)^47,48^ are for tissu-specific expression. *UAS-egr IR* (BL58993)^13^ and *UAS-hep RNAi* (VDRC109277)^49^ are for gene knockdown. *UAS-puc* (BL20627)^50^ is for overexpression.

To starve flies on the protein-restrict diet, the 0–3-day old males were transferred onto protein deficient food (16% sucrose/0.7% argar)^3^ at a density of 20–30 flies per vial. Flies were transferred to fresh vials every 2–3 days. For protein refeeding, starved flies were transferred onto the regular food for one to five days. All flies were reared at 25 °C unless otherwise noted.

### Temperature shift scheme for Gal4/UAS-mediated experiment

For co-overexpression of *DTS5* and *DTS7* by *mef2-Gal4* and knockdown of *egr* by *tara-Gal4*, flies were raised at 18 °C during development and the 0–3-day old adult males were maintained at 29 °C throughout the entire fed or starved/refed stages. To inactivate JNK signaling in cyst lineage, *eyaA3*^*ts*^ > *UAS-hep RNAi* and *eyaA3*^*ts*^ > *UAS-puc* flies were raised at 25 °C during development. Adult flies were maintained at 25 °C during starvation and moved to 29 °C three days before protein refeeding in order to inactivate JNK signaling in the refeeding phase.

### Immunostaining

Immunofluorescence staining of testes was performed as described previously^12^. The following primary antibodies were used: mouse anti-Arm (DHSB, 1:100), mouse anti-Fasciclin III (DSHB, 1:100), rabbit anti-GFP (Invitrogen A11122, 1:250), rat anti-DN-cadherin (DSHB, 1:20), goat anti-Vasa (Santa Cruz dc-13, 1:250), rabbit anti-Vasa (Santa Cruz d-260, 1:500), Rabbit anti-Zfh-1 (1:5,000)^29^. Secondary antibodies conjugated to Alexa 488- (Molecular Probes), Cy3 or Cy5 (Jackson ImmunoResearch) were used at 1:250. Hoechst 33342 was used to stain DNA at 1:500 for 15 min.

### Classification of testis phenotypes

The grading of ectopic Zfh-1 expression and excess early germ cells was described previously^12^. Ectopic Zfh-1 cells are defined as the Zfh-1 positive cells located over 200 μm away from the hub. Level 1, 2, and 3 are defined by the number of ectopic cells less than 50 (level 1), between 50 and 200 (level 2) and more than 200 (level 3). The grade of excess early germ cells were classified into grade 1, grade 2, and grade 3 based on the range of early germ cell accumulation at 200–400 μm, 400–600 μm, and more than 600 μm away from the hub, respectively.

The classification of Egr-GFP levels is based on the specific GFP signal strength and the size of GFP-positive area in the testis sheath. Clear GFP signals in muscle cell cytoplasm throughout the whole testis was classified as “high.” The testes showing Egr-GFP in only some of the muscles or showing relatively weaker Egr-GFP signals were classified as “moderate”.

GSCs are defined as the Vasa-positive cells in contact with hub. CySCs are the Zfh-1 positive cells immediately adjacent to GSCs.

### Feeding assay

Quantification of food intake was based on the uptake of a blue dye (Brilliant Blue R, sigma B7920-10G). A batch of four flies of each genotype was transferred onto fresh food containing blue dye (2.5% w/v) or not at 10 AM for 360 min. Flies were homogenized in 150 μL methanol. Homogenates were centrifuged at 13,000 rpm for 15 min. 140 μL of the clear supernatant were diluted with same volume of distilled water and the absorbance was measured at OD595 nm using spectrophotometer (SpectraMax M2). The flies exposed to regular non-dyed food were used as the baseline for the spectrophotometry.

### CySC recovery rate during refeeding

During the 5-day refeeding period (R1–R5), the CySC recovery rate at R2 is defined as the percentage of CySC increase at R2 relative to R5: (CySC number at R2 − CySC number at S18)/(CySC number at R5 − CySC number at S18).
